# Mechanisms of action of incretin receptor based dual- and tri-agonists in pancreatic islets

**DOI:** 10.1152/ajpendo.00236.2023

**Published:** 2023-09-20

**Authors:** Franco Folli, Giovanna Finzi, Roberto Manfrini, Alessandra Galli, Francesca Casiraghi, Lucia Centofanti, Cesare Berra, Paolo Fiorina, Alberto Davalli, Stefano La Rosa, Carla Perego, Paul B. Higgins

**Affiliations:** ^1^Dipartimento di Scienze della Salute, Università degli Studi di Milano, Milan, Italy; ^2^Unit of Diabetes, Endocrinology and Metabolism, San Paolo Hospital, ASST Santi Paolo e Carlo, Milan, Italy; ^3^Unit of Pathology, Department of Oncology, ASST Sette Laghi, Varese, Italy; ^4^Dipartimento di Scienze Farmacologiche e Biomolecolari, Università degli Studi di Milano, Milan, Italy; ^5^IRCCS MultiMedica, Sesto San Giovanni, Milan, Italy; ^6^International Center for T1D, Pediatric Clinical Research Center Romeo ed Enrica Invernizzi, DIBIC, Università di Milano, Milan, Italy; ^7^Nephrology Division, Boston Children’s Hospital, Harvard Medical School, Boston, Massachusetts, United States; ^8^Division of Endocrinology, ASST Fatebenefratelli-Sacco, Milan, Italy; ^9^Diabetes and Endocrinology Unit, Department of Internal Medicine, IRCCS San Raffaele Scientific Institute, Milan, Italy; ^10^Unit of Pathology, Department of Medicine and Technological Innovation, University of Insubria, Varese, Italy; ^11^Department of Life & Physical Sciences, Atlantic Technological University, Letterkenny, Ireland

**Keywords:** glucagon, glucagon-like peptide 1, glucose-dependent insulinotropic peptide, islets of Langerhans, type 2 diabetes mellitus

## Abstract

Simultaneous activation of the incretin G-protein-coupled receptors (GPCRs) via unimolecular dual-receptor agonists (UDRA) has emerged as a new therapeutic approach for type 2 diabetes. Recent studies also advocate triple agonism with molecules also capable of binding the glucagon receptor. In this scoping review, we discuss the cellular mechanisms of action (MOA) underlying the actions of these novel and therapeutically important classes of peptide receptor agonists. Clinical efficacy studies of several UDRAs have demonstrated favorable results both as monotherapies and when combined with approved hypoglycemics. Although the additive insulinotropic effects of dual glucagon-like peptide-1 receptor (GLP-1R) and glucose-dependent insulinotropic peptide receptor (GIPR) agonism were anticipated based on the known actions of either glucagon-like peptide-1 (GLP-1) or glucose-dependent insulinotropic peptide (GIP) alone, the additional benefits from GCGR were largely unexpected. Whether additional synergistic or antagonistic interactions among these G-protein receptor signaling pathways arise from simultaneous stimulation is not known. The signaling pathways affected by dual- and tri-agonism require more trenchant investigation before a comprehensive understanding of the cellular MOA. This knowledge will be essential for understanding the chronic efficacy and safety of these treatments.

## INTRODUCTION

The binding of endogenous glucagon-like peptide-1 (GLP-1) and glucose-dependent insulinotropic peptide (GIP) to their cognate receptors on pancreatic β cells is responsible for the incretin-effect, that is, the augmented insulin secretion that occurs when glucose is absorbed through the gastrointestinal tract. The insulinotropic effects of these secretagogues occur only at glucose concentrations exceeding normal fasting levels thereby safeguarding against hypoglycemia, and markedly enhancing their utility for the treatment of type 2 diabetes mellitus (T2DM).

The glucagon-like peptide-1 receptor (GLP-1R) is a G-protein-coupled receptor (GPCR) expressed on pancreatic β-, α-, and δ cells. This receptor is internalized in response to GLP-1 binding and triggers a signaling cascade that results in enhanced glucose-stimulated insulin secretion in β cells and reduced glucagon secretion in α cells. The glucose-dependent insulinotropic peptide receptor (GIPR) is also a GPCR and shares ∼42% amino acid sequence homology with the GLP-1R. Its insulinotropic action is also dependent on GIP binding to the extracellular domain of the protein resulting in conformational changes that trigger several intracellular signaling cascades ultimately resulting in enhanced glucose-stimulated insulin secretion. The glucagon receptor (GCGR) is also expressed on islet β and α cells and shares considerable amino acid homology with incretin receptors. GCGR agonism in β cells has been shown to induce insulin secretion (1) whereas glucagon’s actions on α cells lead to further glucagon release in a positive feedback manner (2).

Novel molecules known as unimolecular dual GIPR/GLP-1R agonists have been generated and tested over the past number of years. Interest in developing these molecules was stimulated by in vivo study findings showing that combining GLP-1 signaling with GIP, glucagon (GCG), or both enhanced insulin response beyond that generated by GLP-1 alone. Several of these novel molecules have now entered clinical trials and some have demonstrated favorable outcomes when compared with established GLP-1R agonists. For example, tirzepatide (LY3298176), which is a dual GLP-1R-GIPR agonist, showed superior HbA1c lowering efficacy in patients with T2DM versus GLP-1R agonism alone. In addition, triple agonists that extend to GCGR agonism in the presence of both GLP-1R and GIPR agonism have been developed and are in early-stage clinical trials. In this scoping review, we outline the established cellular mechanisms of action (MOA) while highlighting and discussing additional mechanisms underlying the actions of these novel and therapeutically important classes of peptide receptor agonists (3, 4).

### History of the Incretin System

The history of the incretin system began in 1932 when Belgian physiologist Jean La Barre demonstrated the existence of a substance extracted from the upper intestine that produced hypoglycemia without stimulating exocrine pancreatic secretion in dogs and rabbits. He first named this substance “incrétine” (incretin) and he hypothesized its use to treat diabetes in humans (5). However, the unequivocal demonstration that oral glucose administration resulted in a significantly higher insulin response than intravenous glucose administration, thus showing the insulinotropic activity of the gut, came later, in 1964 (6, 7). Subsequently, other research groups have demonstrated that gastrointestinal factors accounted for at least 50% of insulin secretion (8), and in 1969 the term “enteroinsular axis” was coined to identify the new biological system defining the complex network linking the gut to the endocrine pancreas (9). Then in the early 1970s it was identified the GIP (gastric inhibitory polypeptide) and the acronym was later declined to “glucose-dependent insulinotropic polypeptide” due to its demonstrated insulinotropic effect in humans (10–12). Finally in 1985, GLP-1 (glucagon-like peptide), produced by the pro-glucagon gene and exerting a strong insulin-stimulating effect, was discovered (13). Since then, many intestinal insulinotropic hormone agonists and analogs with beneficial effects beyond glycemic control have been developed, rising to a novel therapy for type 2 diabetes based on the incretin concept (14–18).

## RECEPTORS: TISSUE EXPRESSION AND ESTABLISHED CELLULAR AND WHOLE BODY FUNCTIONS

### GLP-1R

**Figure 1.**
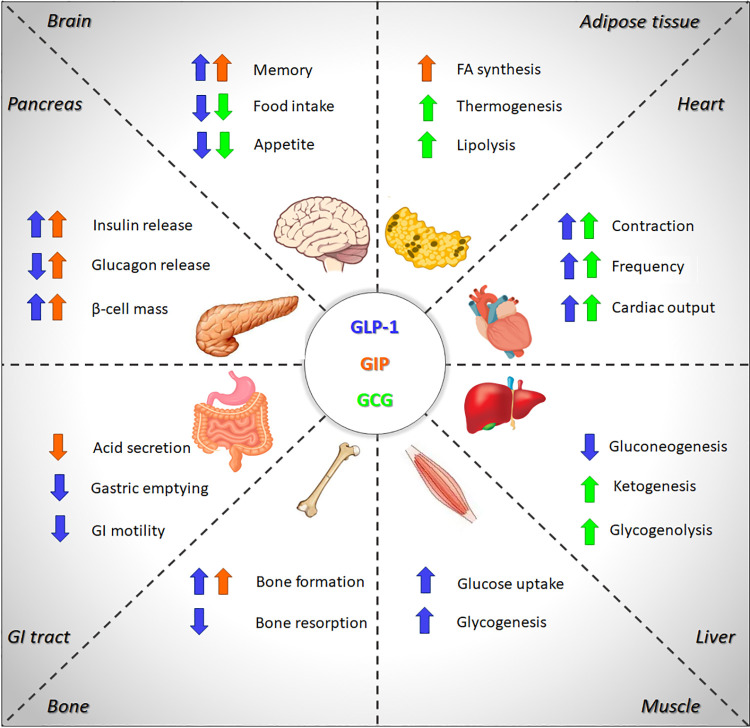
Multi-organ effects of glucagon-like peptide 1 (GLP-1), glucose-dependent insulinotropic polypeptide (GIP), and glucagon (GCG). Arrows indicate the effects of GLP-1 (blue), GIP (orange), and GCG (green) on systems metabolism.

GLP-1 is constitutively secreted from enteroendocrine L cells (localized in ileum and colon) at low concentrations during fasting, with secretion significantly stimulated following direct and indirect L cell stimulation in the postprandial state. GLP-1 was initially shown to increase insulin gene expression and stimulate insulin secretion (19, 20). Further studies spanning the past three decades have uncovered multiple additional actions of GLP-1 including lowering food intake, inhibiting glucagon secretion, and reducing gastric emptying (21) (Fig. 1). In addition, GLP-1-mediated metabolic effects include increased glucose uptake and glycogenesis in the skeletal muscle (22) and the inhibition of gluconeogenesis in the liver (23). Besides these metabolic effects, GLP-1 also shows chronotropic and inotropic effects as suggested by the impaired left ventricle contractility and diastolic dysfunction in GLP-1R-deficient mice (24). GLP-1 signaling requires binding to its cognate receptor GLP-1R (25). The GLP-1R is a class B GPCR and is expressed broadly across several tissues and organs including pancreatic islet cells, the heart, the central and peripheral nervous systems, and vasculature cells. Expression of the GLP-1R protein has been confirmed in many tissues in several different species, such as rodents, monkeys, and humans (26–28). Fluorescent peptides have recently been used to locate GLP-1R ligand binding sites and have identified subtypes of GLP-1R-expressing pancreatic α cells (29). The GLP-1R is a class B GPCR and therefore contains an extracellular N-terminal domain for ligand recognition and binding. In addition, it contains an intracellular C-terminal with a seven transmembrane α-helix domain interacting with the cellular membrane where each transmembrane residue is connected by three intracellular loops and three extracellular loops. Class B GPCRs typically bind ligands in a two-domain model, in which the extracellular domain binds to the C-terminal end of the ligand first, enabling a second interaction to occur between the N-terminus of the ligand and the seven transmembrane domains of the receptor (30).

### GIPR

GIP, a 42 amino acid protein, is constitutively secreted from K cells (localized in duodenum and jejunum) during fasting and shows a marked postprandial increase in secretion (31–33). GIP was first identified thanks to its ability to inhibit gastric acid secretion and after its discovery, several studies have unveiled the multi-organ effects of GIP (34). The systemic effects are mediated by its binding to the GIP receptor (GIPR), a class B GPCR, broadly expressed across several tissues. Indeed, GIPRs are expressed in the pancreas (24), the adipose tissue (35), the bone (36, 37), and the stomach (32); GIPRs were also detected in several brain regions including cerebral cortex, hippocampus, and olfactory bulb (38, 39).

As an incretin, GIP enhances glucose-stimulated insulin secretion and indirectly modulates glucagon secretion (40). Despite their similarities, the effects of GIP and GLP-1 on glucagon release are opposite. Indeed, GIP stimulates glucagon secretion via an increase of intracellular cyclic adenosine monophosphate (cAMP) levels as revealed by studies performed in isolated rat islets (41). This enhancement of glucagon secretion, which was also confirmed in healthy subjects and subjects with T2DM, hinders the clinical use of GIP agonists for diabetes treatment (24, 42).

Besides its insulinotropic effects, GIP has a critical role in fat accumulation by increasing the activity of lipoprotein lipase (LPL) expressed in adipocytes (24, 35) and stimulates bone formation by inhibiting osteoclast apoptosis, as suggested by the presence of thinner bone trabeculae in GIPR-deficient mice (36). GIP-transgenic mice also showed improved memory, which is probably related to the enhancement of neurogenesis (43). In line with this hypothesis, it has been showed that GIP infusion promotes neuronal progenitor proliferation in the dentate gyrus, whereas GIP-deficient mice showed worsening of memorial tasks due to decreased neurogenesis (44) (Fig. 1).

### GCGR

Glucagon hyperglycemic actions were first described over one hundred years ago (45, 46). This hormone binding to its receptors cooperates with insulin to regulate blood glucose levels (34). Hyperglycemic effects of glucagon are mainly attributed to its ability to control hepatic glucose metabolism (47). Glucagon strongly induces glycogenolysis and inhibits glycogenesis in the liver, ensuring a constant supply of glucose (48–50).

Besides the well-established role of glucagon in maintaining glycemia, several studies have uncovered multiple additional actions of glucagon in the brain (51), the liver (52), the heart (53), and the adipose tissue (51).

Glucagon receptors were first identified in the rat brain almost 40 years ago (54), suggesting a potential role of this hormone in regulating brain functions. In accordance, it has been reported that glucagon decreases food intake, appetite, and promotes weight loss in rodents (55, 56) and humans (57–59). Although the effect of glucagon on body weight is so far established, some studies did not observe changes in food intake after glucagon administration, thus suggesting appetite-independent mechanisms involved in the glucagon-mediated control of body weight (34). In line with this hypothesis, a rapid increase in metabolic rate after a single subcutaneous administration of glucagon in rats has been demonstrated (60). Glucagon enhances metabolic rates by stimulating oxygen consumption in brown adipose tissue (BAT), as revealed by increased BAT temperature in rats (61, 62). Glucagon also exerts lipolytic effects on the white adipose tissue by inhibiting lipogenesis and stimulating lipolysis (34). The lipolytic effect is mediated by the activation of the hormone-sensitive lipase (HSL) in the adipocytes (63) and it is amplified by indirect mechanisms including the secretion of growth hormone (64), cortisol (65), and epinephrine (66).

Glucagon effects on lipid metabolism are not only restricted to the adipose tissue but also include an increase of ketogenesis in the liver (67). Indeed, glucagon stimulates the formation of ketone bodies by constantly supplying nonesterified fatty acid to the liver (68, 69) and blocking the hepatic glycolytic pathway (70). These result in enhanced fatty acid oxidation in the mitochondria and potentiation of hepatic ketogenesis (34, 70). Glucagon also enhances cardiac output by binding to its receptors, leading to the activation of adenylate cyclase (AC) and subsequently increase of cAMP levels in the myocardium. The chronotropic and inotropic effects of glucagon are extremely rapid with a peak in 5 min and a duration of 20 min after its administration (34, 53).

## INTRACELLULAR SIGNALING PATHWAYS IN THE ISLETS OF LANGERHANS

GLP-1R, GIPR, and CGCR are primarily expressed in islets of Langerhans, on β- and α- and δ cells, where they affect hormone secretion and control endocrine cell proliferation and survival.

### Beta Cells

**Figure 2.**
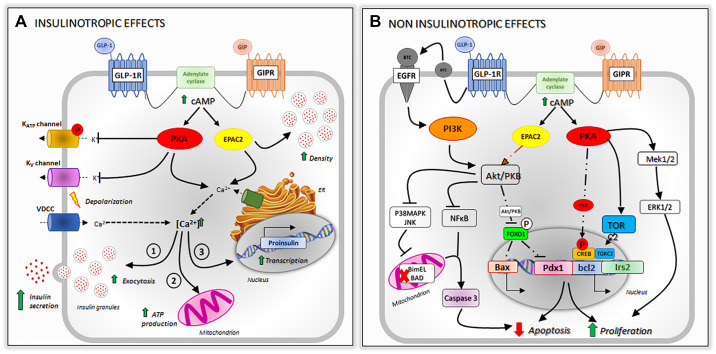
Effects of glucose-dependent insulinotropic polypeptide (GIP) and glucagon-like peptide 1 (GLP-1) on pancreatic β-cells. *A*: insulinotropic effects. GIP and GLP-1 bind to their receptors [glucose-dependent insulinotropic peptide receptor (GIPR) and glucagon-like peptide-1 receptor (GLP-1R), respectively] and activate the adenylate cyclase. The subsequent elevation in intracellular cyclic adenosine monophosphate (cAMP) activates protein kinase A (PKA) and exchange protein activated by cAMP-2 (EPAC2). PKA induces the closure of K^+^_ATP_ channel, facilitating membrane depolarization, and K^+^_V_ channels, leading to the prolongation of action potentials. Membrane depolarization leads to the opening of voltage-gated Ca^2+^-channels (VDCC), allowing the elevation of intracellular Ca^2+^ that promotes insulin release through different mechanisms: *1*) the fusion of insulin granules with the plasma membrane, *2*) ATP production within the mitochondria, and *3*) the transcription of proinsulin gene. PKA and EPAC2 induce Ca^2+^ release from intracellular stores, strengthening Ca^2+^-mediated exocytosis. EPAC2 also increases the density of insulin containing granules near to the plasma membrane. *B*: noninsulinotropic effects. GIP induces the activation of adenylate cyclase and the elevation of intracellular cAMP leading to the activation of PKA and EPAC2. PKA inhibits AMPK signaling promoting the translocation of transducer of regulated cAMP response element-binding protein (CREB) (TORC2) into the nucleus, where it binds to P-CREB and promotes the transcription of the antiapoptotic gene Bcl2. The proliferative and prosurvival effects of GLP-1 are mediated through the transactivation of the epidermal growth factor receptor (EGFR) that, in turn, induces the activation of the PI3K/Akt/PKB signaling. It causes the phosphorylation of the nuclear transcription factor (Foxo1) which leads to its translocation outside the nucleus, limiting the activity of proapoptotic pathways. Concomitantly, PI3K inhibits the NFkB and P38MAPK/JNK pathways which reduce the activation of Caspase 3 and thus, the β-cell apoptosis. ER, endoplasmic reticulum.

Studies demonstrating the insulinotropic and glucose-lowering effects of GLP-1 in islet β cells combined with the hyperglycemic effects of GLP-1R antagonism stimulated the search of GLP-1R agonists for the treatment of T2D (71). Crucially, the glucose-lowering actions of GLP-1 persist in patients with T2D. GLP-1R activation in the β cell leads to insulin gene transcription and translation, and the potentiation of glucose-stimulated insulin secretion (insulinotropic effects). Longer-term effects of GLP-1R activation include enhanced proliferation and neogenesis of β cells combined with cytoprotective effects (noninsulinotropic effects) (72–81) (Fig. 2).

### GLP-1R Signaling

It is known that signaling through the canonical GLP-1R is mediated by heterotrimeric G proteins. These G proteins contain an independent Gα subunit and a Gβ/γ dimer subunit. Agonist-activated GPCRs facilitate guanine triphosphate (GTP) production and subsequent GTP binding to the G-protein induces separation of the Gα and Gβγ subunits that can then activate downstream signaling proteins (82). GLP-1 was first shown to activate adenylate cyclase in central nervous system (CNS) tissue (83). That ligand-activated GLP-1R interacts with the Gαs subunit and stimulates adenylate cyclase to produce cyclic adenosine monophosphate (cAMP) is established. Increased cAMP promotes activation of both protein kinase A (PKA) and the exchange protein activated by cAMP-2 (EPAC2) (84). Both pathways synergically promote insulin secretion by increasing the Ca^2+^ influx and hence insulin granule exocytosis. PKA directly phosphorylates sulfonylurea receptor 1 (SUR1), a regulatory subunit of K_ATP_ channels and voltage-dependent K^+^ channels, thus increasing membrane depolarization and the activation of voltage-gated Ca^2+^ channels. EPAC2 activation may promote insulin granules exocytosis by different mechanisms, including direct interaction with SUR1 and membrane depolarization, calcium mobilization from intracellular stores, and modification of insulin granules priming (85, 86). Noninsulinotropic effects of GLP-1R activation have also been described. Indeed, the GLP-1R-cAMP-PKA axis promotes β-cells proliferation, through the activation of the transducer of regulated cAMP response element-binding protein (CREB) (TORC2) and cAMP response element-binding protein (CREB) and IRS2 gene expression (87, 88). In addition, the activated CREB also promotes β cell survival, increasing *Bcl-2* activity, and inhibiting the proapoptotic *Bax* (89). Proliferative and antiapoptotic actions of GLP-1 are also mediated by the PI3K/Akt axis, via transactivation of the epidermal growth factor receptor (EGFR), an event linked to GLP-1R via activation of c-Src and the production of EGF-like endogenous ligands (90). Although not well-established, GLP-1R agonism may activate other G-protein subunits and alternative downstream signaling pathways as shown in some cell lines (91–94). An overview of both established and putative GLP-1R signaling pathways is given in Table 1.

**Table 1.** Overview of GLP-1R, GIPR, and GCGR G-protein-mediated signaling pathways in β cells

Receptor	Pathways	Cellular Processes
GLP-1R	Gα_s_ → Adenylate Cyclase → cAMP → PKA Gα_s_ → Adenylate Cyclase → cAMP → PKA → AMPK Gα_s_ → Adenylate Cyclase → cAMP → EPAC2 Gα_q_ → PKC Gα_q_ → MAPK	Proinsulin transcription, insulin granule exocytosis, proliferation, antiapoptosis Proinsulin transcription, insulin granule exocytosis. Insulin granule exocytosis. Proliferation
GIPR	Gα_s_ → Adenylate Cyclase → cAMP → PKA Gα_s_ → Adenylate Cyclase → cAMP → MAPK→ERK1/2 Gα_s_ → Adenylate Cyclase → cAMP → EPAC2 → Akt/PKB	Proinsulin transcription, insulin granule exocytosis, proliferation. Proliferation. Antiapoptosis.
GCGR	Gα_s_ → Adenylate Cyclase → cAMP → PKA	Proglucagon transcription, glucagon granule exocytosis, proliferation. Antiapoptosis.

Glucagon-like peptide-1 receptor (GLP-1R) activation also activates the epidermal growth factor receptor (EGFR) via a non-G protein coupled mechanism involving c-SRC kinase leading to activation of PI3K, AKT/PKB, and phosphorylation of FOXO1 with subsequent antiapoptotic effects (86). GLP-1R internalization occurs via the activation of β-Arrestin which also activates ERK1/2 and may contribute to proliferation. cAMP, cyclic adenosine monophosphate; EPAC2, exchange protein activated by cAMP-2; GCGR, glucagon receptor; GIPR, glucose-dependent insulinotropic peptide receptor; MAPK, mitogen-activated protein kinase; PKA, protein kinase A.

**Figure 3.**
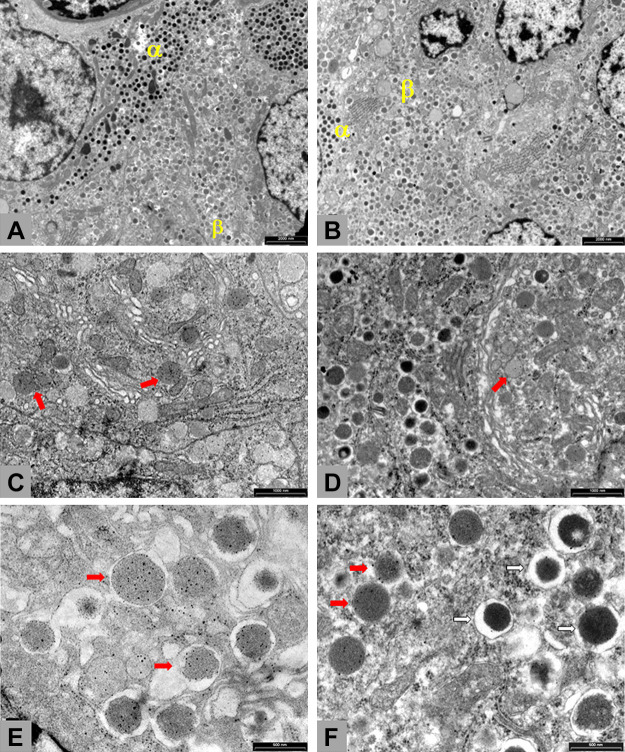
Transmission electron microscope (TEM) images of islet of Langerhans from saline/control (*A*, *C*, and *E*) and exenatide-treated pancreas (*B*, *D*, and *F*) (G. Finzi, S. La Rosa, and F. Folli, unpublished observations). Pancreatic specimens of four saline-treated and four exenatide-treated baboons were fixed in 2% paraformaldehyde and 2% glutaraldehyde (Karnovsky fixative), post-fixed in 1% osmium tetroxide, and embedded in Epon.Araldyte. Thin sections were counterstained with uranyl acetate and lead citrate and observed with a Philips/Morgagni/Thermo Fisher Scientific electron microscope (FEI Thermofisher Company, Eindhoven, the Netherlands). *A* and *B*: exenatide-treated pancreases show endocrine cells healthy and well granulated. *C* and *D*: TEM from control and exenatide-treated pancreas immunostained with anti-proinsulin antibodies. For ultrastructural immunocytochemistry, the sections were pretreated with sodium metaperiodate for 30 min, then placed onto a drop of 1% ovoalbumin for 5 min, transferred onto a drop of anti-proinsulin (monoclonal mouse anti-proinsulin DSHB, Gentofte, Denmark) diluted 1:10 overnight, then after rinses transferred onto a drop of 18 nm colloidal gold-AffiniPure (Jackson Immunoresearch, West Grove, PA) goat antimouse diluted 1:20 for 1 h, and, after rinses, counterstained. In controls experiments, primary antibodies were omitted. After exenatide treatment, β cells immature granules, showing a homogenous gray content and proinsulin labeled (red arrows), are less represented than in control pancreas. *E* and *F*: TEM from control and exenatide-treated animals immunostained with anti-proinsulin (18 nm colloidal gold) and anti-insulin (12 nm colloidal gold) antibodies. After sodium metaperiodate, thin sections were placed onto a drop of 1% ovoalbumin for 5 min, transferred onto a drop of anti-proinsulin (monoclonal mouse anti-proinsulin DSHB, Gentofte, Denmark) diluted 1:10 overnight, then after rinses transferred onto a drop of anti-insulin (polyclonal guinea pig anti-insulin, Dako, Glostrup, Denmark) diluted 1:50 overnight, after subsequent rinses transferred onto a mixture of 18 nm colloidal gold-AffiniPure (Jackson Immunoresearch, West Grove, PA) goat anti-mouse diluted 1:20 and of 12 nm colloidal gold-AffiniPure (Jackson Immunoresearch, West Grove, PA) donkey anti-guinea pig diluted 1:20, and finally counterstained after rinses. Controls experiments were done with primary antibodies omission. After saline treatment, β cells granules are predominant immature, showing homogenous gray matrix (red arrows) and containing proinsulin. After exenatide treatment β cells exhibit numerous mature granules, characterized by dense cores and peripheral clear halos (white arrows) and insulin content, beyond immature granules.

GLP-1 and the GLP1 receptor agonist, exendin 4 prevents β cell death and augments β cell proliferation in animal models (73, 75, 97). Stimulation of GLP-1R signaling upregulates insulin synthesis and secretion while also ameliorating endoplasmic reticulum stress in β cells via cAMP-dependent effects (75, 98) (Fig. 3). These findings are consistent with the notion that GLP-1R agonism over time preserves functional β cell mass during therapy (up to 24 mo) with GLP-1R agonists and improved β cell function is achieved in patients with T2D. In human subjects with T2D, liraglutide provided robust enhancement of β-cell function that is sustained over 48 wk in early T2D but it was lost upon 2 wk of therapy cessation (99).

Improvements in β cell function and disposition index were found in a 3-yr follow-up study of subjects with T2D treated with multiple daily injections of exenatide after 4 wk of drug discontinuation, however the exenatide treatment resulted in a weight reduction that complicates the interpretation of these findings (100). Despite current limitations in imaging β-cell mass in humans, available data in nonhuman primates (baboons) support the concept that treatment with GLP-1R agonists will lead to a lasting improvement in human β cell function through persistent changes in β cell mass (73, 75).

Preclinical data on the role of GLP-1R signaling in the maintenance and expansion of functional β cell mass are somehow difficult to reconcile with available clinical data. In response to GLP-1, the β cells of young mice proliferate whereas the β cells of older mice show a generalized loss of proliferative capacity (101, 102), moreover these also show attenuated proliferation in response to GLP-1R agonism (101).

Older human cells also lose basal and GLP-1-stimulated proliferative capacity, regardless of the relative levels of mRNA transcripts, which remain comparable with those of young human islet β-cells (103). The proliferative action of GLP-1R agonists in young human islets has been related to the functional integrity of calcineurin/nuclear factor activated T cell (NFAT) signaling, since, when NFAT has been inhibited via FK506, the action of exentide-4 on β cell proliferation (103). Furthermore, in islets from adult human donors (20 yr of age and older) the NFAT signaling pathway was also impaired (103). These findings should be taken into account when considering the use of GLP-1R agonists for the therapeutic increase of human β-cell mass.

### GIPR Signaling

The 42 amino acid mature GIP binds to and activates its cognate receptor GIPR on the β cell surface. Consistent with GLP-1R activation, ligand binding to GIPR activates Gαs that in turn activates adenylate cyclase leading to cAMP production. Increased cAMP activates PKA and EPAC. cAMP and PKA also activate a series of proteins including the mitogen-activated protein kinase (MAPK) cascades, and phosphorylates ERK1/2, which regulates genes involved in proliferative and antiapoptotic processes (104). The GIPR activation of PKA leads to insulin secretion by the same mechanisms (potassium channel closure-mediated membrane depolarization) as described for the GLP-1R. GIPR activation can also promote noninsulinotropic actions, such as controlling pancreatic β cell proliferation and survival (105, 106). In addition, in GIPR and GLP-1R signaling, PKA activation is known to inhibit AMPK, leading to the transfer of TORC2 into the nucleus (107). In the nucleus, CREB and TORC2 form a complex promoting the transcription of the antiapoptotic gene bcl2 (107). Furthermore, activation of Akt/PKB/PI3K causes phosphorylation of the nuclear transcription factor (Foxo1), which then inactivates molecules to limit the activity of proapoptotic pathways (107). An overview of both established and putative GIPR signaling pathways is given in Table 1.

### Intracellular Signaling Pathways in β Cells: Overlap and Synergy

There is considerable overlap in the signaling pathways activated by ligand binding to the GLP-1R and GIPR in β cells (Fig. 2). Both receptors activate adenylate cyclase resulting in increased intracellular cAMP, activating PKA and EPAC2 pathways that result in insulin production and release, cellular proliferation, and antiapoptotic effects. Both receptors are desensitized by recruitment of β-arrestins, followed by receptor internalization, recycling, and inactivation of Gαs.

Several differences also exist. First, the GLP-1R can be internalized both by β-arrestins and by Gαq and internalization is influenced by several ligands. In contrast, GIPR internalization is not that readily influenced by novel ligands and is dependent entirely on arrestins (108). These β-arrestins play a key role in GIPR desensitization by blocking the Gαs proteins and in receptor trafficking by internalization and recycling. Second, activation of the GIPR in β cells has been shown to result in MAPK-induced signaling pathways, which is not seen for the GLP-1R. Third, GLP-1R activation promotes EGFR signaling leading to proliferation and antiapoptosis (24), this has not been demonstrated for GIPR activation.

### GIP and GLP Signaling in α Cells

GLP-1 and GIP action on α cells has been also described. GLP-1 stimulation also lowers glycemia through suppression of glucagon secretion by α cells (109). A subset of α cells in culture showed GLP-1R expression (110) and α-cell specific GLP-1R knockout (KO) mice exhibit mild glucose intolerance and increased glucagon secretion in response to glucose challenging compared with wild-type animals, suggesting a possible direct effect of GLP-1 on this cell (111). It is also possible that GLP-1 exerts an indirect effect on α cells. Indeed, several β cell-secreted molecules have been shown to inhibit glucagon secretion, including insulin, zinc, and γ-aminobutyric acid, and could theoretically contribute to the inhibition of GLP-1-dependent α-cell secretory activity (21). Furthermore, as GLP-1 stimulates islet somatostatin secretion directly though the canonical GLP-1R, expressed on δ-cells, it can also be possible that is the somatostatin that directly inhibits glucagon secretion by binding to its receptor in the α cell (112). Evidence in this direction comes from experiments in isolated perfused rat pancreas, where co-infusion with a somatostatin receptor 2 antagonist or treatment with anti-somatostatin antibodies completely abolished the GLP-1-mediated suppression of glucagon secretion (112). Contrary to GLP-1, GIP stimulation promotes glucagon secretion by α cells (97), an effect mediated by the direct action of the peptide on the GIPR, expressed by these cells (113, 114). Functionally, GIPR activation increases cAMP/PKA signaling pathways, resulting in cell depolarization, enhanced intracellular Ca^2+^ concentration and glucagon secretion (115, 116). Interestingly, GIPR activity in the α cell seems to be glucose dependent, because GIP perfusion in the intact rat pancreas stimulates glucagon secretion only at low glucose (4.4 mM) and not at postprandial glucose concentrations (8.9 mM) (41). GIP-mediated increase in circulating glucagon concentrations has been detected also in healthy humans, similar to preclinical studies this effect is glucose dependent, as it has been shown to occur only under hypoglycemia (113, 117).

## RATIONALE FOR RECEPTOR CO-AGONISM

### Mechanisms of Action of Receptor Co-Agonism: Additive or Synergistic Interaction Effect

Initial synergy between GLP-1 and GIP was shown in rat islets (118) over 30 yr ago. Both GLP-1 and GIP are secreted in response to food ingestion as part of the incretin effect (97). After secretion, both peptides are rapidly degraded by the enzyme dipeptidyl peptidase IV and removed by the kidneys (119). In physiological conditions, GLP-1 and GIP are responsible for 70% of insulin release after an oral glucose tolerance test (OGTT). The insulin secretion is glucose-dependent and occurs only in the presence of high glucose levels (120). In the condition of chronic hyperglycemia and/or insulin resistance (e.g., DM2), GLP-1 levels are reduced as part of the reduction/disappearance of incretin effect that characterizes these conditions. Differently from GLP-1, GIP levels are not reduced, and the poor metabolic effect of GIP seen in these cases might depend on a resistance phenomenon (121, 122). Furthermore, as earlier reported, GLP-1 and GIP have essentially a similar stimulating effect on the β cell, but they have a different effect on the α cell. In fact, while GLP-1 constantly inhibits glucagon secretion, GIP has a bi-directional effect: it stimulates glucagon secretion only under conditions of normoglycemia and hypoglycemia, but not in the presence of hyperglycemia. This may mitigate and protect against further postprandial hyperglycemic stimulation in pre-existing hyperglycemic conditions (e.g., diabetes mellitus) (123). Besides the insulinotropic effects, GLP-1 and GIP show metabolic and nonmetabolic effects as their receptors are extensively expressed in the body: for GLP-1 at the pancreas, heart, kidneys, stomach, lungs, and central nervous system; for GIP at the pancreas, adipose tissue, bone, intestine, heart, pituitary gland, adrenal gland, and brain (26, 39). This extensive receptor distribution may account for the antiatherosclerotic, endothelium-stabilizing, antiapoptotic, anti-inflammatory, antiobesity, and neurotrophic effects shown by GLP-1 in type 2 diabetes, obese, and dysmetabolic subjects (124). In fact, GLP-1 receptor agonists have demonstrated effective hypoglycemic action, anti-inflammatory and antiapoptotic effects, and counteracting atherosclerosis and its complications. These mechanisms of action have subsequently demonstrated clinically significant protective effects at the cardiac and renal levels and also on the liver (125–132). In addition, GLP-1 binding to specific hypothalamic receptors (arcuate nucleus, dorsomedial nucleus, and ventromedial nucleus), via POMC/CART and NPY/AgRP, increases satiety sensation and reduces food cravings (133). GLP-1 also induces satiety peripherally because it slows gastric emptying. This sometimes results in nausea, which in severe cases can lead to discontinuation of the drug (134).

Regarding GIP, the main metabolic actions involve an insulinotropic and antiapoptotic effect at the pancreatic level, the stimulation of fat deposition in adipocytes, the central control of food intake with weight loss, and a trophic action on bone (135, 136). In turn, the cardiovascular effects of GIP are more conflicting and difficult to interpret. In fact, the actions of GIP on endothelium, on atherosclerotic plaque, and inflammation are not unidirectionally protective and seem very complex. More studies are needed in this direction (137).

Regarding glucagon, in addition to its known hyperglycemic effect, the hormone has demonstrated a number of systemic metabolic effects that make it of interest in the therapy of obesity and diabetes (138). On the other hand, GLP-1 and glucagon share a common origin from proglucagon, so a role of glucagon in regulating metabolism has been hypothesized (139). The permeability of the blood-brain barrier to glucagon and the discovery of marked immunoreactivity for glucagon in the arcuate nucleus (ARC) and brain system subsequently delineated the role of glucagon as a modulator of food intake (140). Specifically, administration of intracerebroventricular glucagon in animals and intravenously in humans has been shown to significantly reduce food intake. Studies have shown that the anorectic action of glucagon would occur in ARC by activating PKA/CaMKKb/AMPK-dependent pathways (141). Glucagon also stimulates increased energy expenditure in rodents and humans, and this may realize a synergistic effect with the anorectic effect in the context of controlling food intake and weight (142, 143). Therefore, the action of glucagon, in addition to that of GIP and GLP-1, could result in additional metabolic benefits, such as increased energy expenditure and improved metabolic performance.

### Rationale for Receptor Dual and Tri-Agonism

Given the multi-organ effects of these gastrointestinal hormones, which act centrally and peripherally, the rationale of using dual- and tri-agonism resides in the large effect on weight loss and reduces obesity-related metabolic risks leading to a significant improvement of the diabetic conditions. Specifically, the GIP-GLP-1 co-agonism allow to reach a better glycemic control and weight reduction in subjects with obesity and diabetes (15, 144), as both these gastrointestinal hormones account for meal-mediated insulin secretion (145), have an insulinotropic action, and act centrally and peripherally to reduce appetite and stimulate satiety. GLP-1 also induces satiety peripherally because it slows gastric emptying.

The rationale for GLP-1/glucagon co-agonism is to achieve weight loss in combination with good glycemic control and energy equilibrium, as glucagon induces energy expenditure, increases lipid metabolism, and inhibits food intake (63, 146, 147). Balance of GLP-1 and GCG receptors potency within a co-agonist can predict clinical effects while minimizing adverse effects (i.e., nausea if GLP-1 action prevails or increased hepatic glucose production if glucagon action prevails) (139).

In addition, simultaneously engaging GLP-1, GIP, and GCG receptors, using GLP1-GIP-GCG receptors tri-agonist, allows multiple central and peripheral effects crucial for achieving and maintaining metabolic balance. These combined actions encompasses a wide range of effects and positive impact on weight reduction, food intake, glycemia control, and energy expenditure (148).

Moreover, it could be relevant that the effects of GLP1-RA (GLP-1 receptor agonist) on the liver are well documented in previous work (149).

**Figure 4.**
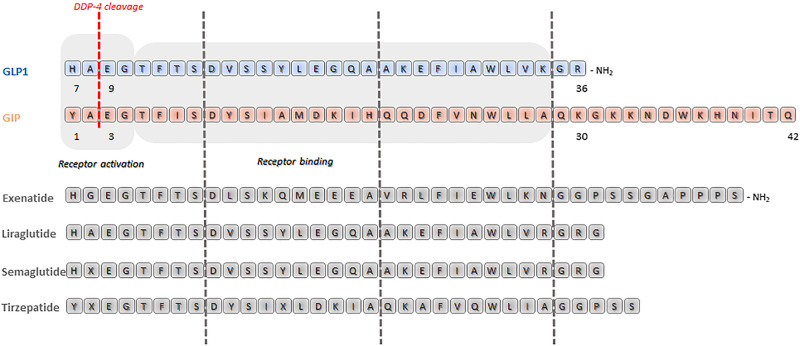
Amino acid sequence homology in glucagon-like peptide 1 (GLP-1, blue), glucose-dependent insulinotropic polypeptide (GIP, orange), and glucagon-like peptide-1 receptor (GLP-1R) agonists, and dual incretin agonists (gray). The DDP-4 cleavage site is reported in red.

An overview of existing dual- and tri-agonists under development for targeting of β cell function is reported in Fig. 4.

## EFFICACY OF GLP-1R-GIPR DUAL-AGONISM IN CLINICAL STUDIES

The first GLP-1-GIP dual agonist developed was NN9709 (formerly MAR709 and RG7697). However, although it demonstrated in phase II studies the ability to reduce weight and blood glucose in diet-induced obesity mice, its development was discontinued given the efficacy of semaglutide 2.4 mg in phase III studies (150). Subsequent studies were on tirzepatide, a once-weekly subcutaneous injectable peptide sequence-derived from native GIP, with agonist activity for both GIP and GLP-1 receptors (151). The safety and efficacy of tirzepatide were tested in the SURPASS phase 3 clinical trial program.

### Surpass Program

1.  In the multicentric double-blind phase 3 study, 478 subjects with diabetes and obesity were randomized to tirzepatide 5 mg, tirzepatide 10 mg, tirzepatide 15 mg, or placebo. At 40 wk, tirzepatide induced a dose-dependent weight loss ranging from 7.0 to 9.5 kg. The most frequent adverse events with tirzepatide versus placebo were mild to moderate and transient nausea (12–18% vs. 6%), diarrhea (12–14% vs. 8%), and vomiting (2–6% vs. 2%). HbA1 decreased from baseline by 1.87% with tirzepatide 5 mg, 1.89% with tirzepatide 10 mg, and 2.07% with tirzepatide 15 mg versus placebo (152).
2.  In an open-label, 40-wk, phase 3 study, 1,879 patients with type 2 diabetes were randomly assigned to receive tirzepatide at a dose of 5 mg, 10 mg, or 15 mg or semaglutide at a dose of 1 mg. Tirzepatide was superior to semaglutide in both reducing HbA1 (estimated mean change from baseline in the glycated hemoglobin −0.15 percentage points, −0.39 percentage points, and −0.45 percentage points at a dose of 5 mg, 10 mg, or 15 mg, respectively; *P* < 0.05 for all comparisons) and inducing weight loss (mean estimated treatment difference, −1.9 kg, −3.6 kg, and −5.5 kg, respectively; *P* < 0.001 for all comparisons) (153).
3.  In a phase 3 study enrolling 1,444 subjects evaluated at 52 wk, the effects on glycemia and body weight of once-weekly tirzepatide (5, 10, and 15 mg) versus once-daily titrated insulin degludec in patients with type 2 diabetes in inadequate glycemic control with a stable dose of metformin with or without SGLT2 inhibitors. The reductions in HbA1c at *week 52* were 1.93% for tirzepatide 5 mg, 2.20% for tirzepatide 10 mg, and 2.37 tirzepatide 15 mg compared with 1.34% for insulin degludec. In all three tirzepatide groups, the proportion of participants achieving an HbA1c of less than 7·0% was significantly greater compared with insulin degludec (82%–93% vs. 61%). From a starting bodyweight of 94.3 kg, all three tirzepatide doses decreased bodyweight (−7.5 kg to −12.9 kg), whereas insulin degludec increased bodyweight by 2.3 kg (154).
4.  In a multicenter phase 3 study, 2,002 subjects with type 2 diabetes were randomized to receive tirzepatide at a dose of 5 mg, 10 mg, or 15 mg or glargine (100 U/mL). The study was for 52 wk, and the cardiovascular safety of tirzepatide versus glargine was the prespecified objective. The MACE-4 events (cardiovascular death, myocardial infarction, stroke, hospitalization for unstable angina) were not increased on tirzepatide compared with glargine {hazard ratio 0.74 [95% confidence interval (CI) 0.51–1.08]} (155).
5.  A 40-wk multicenter phase 3 study compared the efficacy of tirzepatide (doses of 5 mg, 10 mg, 15 mg) or placebo in addition to glargine (100 U/mL) in subjects with inadequately compensated diabetes with and without metformin. At the doses of 10 mg and 15 mg tirzepatide showed significantly greater change in HbA1c from baseline at *week 40* versus placebo (10 mg: difference, −1.53% [97.5% CI, −1.80% to −1.27%]; *P* < 0.001; 15 mg: difference, −1.47% [97.5% CI, −1.75% to −1.20%]; *P* < 0.001) (156).

### Other Studies

A multicenter post hoc study investigated the effects of tirzepatide (1, 5, 10, and 15 mg) and dulaglude (1.5 mg) versus placebo on markers of β cell function and insulin sensitivity in 360 subjects with type 2 diabetes. After 26 wk, proinsulin/insulin and proinsulin/C-peptide ratios were significantly reduced with tirzepatide 10, and 15 mg compared with dulaglutide and placebo (*P* ≤ 0.007). In addition, tirzepatide 10 mg significantly reduced HOMA2-IR (Homeostatic Model Assessment for Insulin Resistance) compared with dulaglutide and placebo (*P* = 0.004) (157).

In a double-blind phase study, 2,539 subjects with body mass index (BMI) > 27 with at least one weight-related complication excluding diabetes were randomized to receive tirzepatide at doses of 5 mg, 10 mg, 15 mg, or placebo. The end point was the percentage of weight reduction at 72 wk in the two groups. At the end of the study the mean change in weight was −15.0% (95% CI, −15.9 to −14.2) in the 5 mg weekly dose group, −19.5% [95% CI, −20.4 to −18.5] in the 10 mg dose group, and −20.9% [95% CI, −21.8 to −19.9] in the 15 mg dose compared with −3.1% [95% CI, −4.3 to −1.9] in the placebo group. Treatment interruptions adverse events related (mainly nausea, diarrhea, and constipation) occurred in 4.3%, in 7.1%, and in 6.2% with 5 mg, 10 mg, and 15 mg of tirzepatide, respectively, and 2.6% with placebo (158).

A phase Ia study (NCT03175211) investigated single rising doses (SRDs) of BI 456906 in 24 males with a body mass index (BMI) of 20–<30 kg/m^2^. A phase Ib study (NCT03591718) investigated multiple rising doses (MRDs) of BI 456906 (escalated over 6 [Part A] or 16 [Part B] wk) in 125 adults with a BMI of 27–40 kg/m^2^ versus placebo in healthy volunteers and people with overweight/obesity find BI 456906 produced a placebo-corrected body weight loss of 13.8% (*week 16*), highlighting its potential to promote clinically meaningful body weight loss in people with overweight/obesity (159).

## EFFICACY OF GLP-1R-GCGR DUAL-AGONISM IN CLINICAL STUDIES

In a multicenter placebo-controlled study, dual GLP-1 and glucagon receptor agonist cotadutide, at doses of 100 μg, 200 μg, and 300 μg, was compared with placebo and liraglutide 1.8 mg in 834 participants. All drugs were administered subcutaneously. The study lasted 54 wk, and liver and metabolic parameters were evaluated in subjects with overweight/obesity and type 2 diabetes. All doses of cotadutide and liraglutide showed a significant reduction in A1c with respect to the placebo (*P* < 0.001). No significant differences were observed between cotadutide and liraglutide in A1c reduction. Cotadutide 300 μg resulted in a statistically superior percent in weight reduction than liraglutide and placebo (*P* = 0.009 and *P* < 0.001 respectively). AST level was reduced with cotadutide 200 μg and 300 μg compared with liraglutide (*P* = 0.023). At liver fibrosis, noninvasive scoring system based on laboratory test (FIB-4 and NFS) reductions in FIB-4 at cotadutide doses 200 mg and 300 mg versus placebo (*P* = 0.032 and *P* = 0.004, respectively) were observed. A significant reduction in NFS was seen with cotadutide 300 mg versus placebo (*P* = 0.010). Gastrointestinal disorders, including diarrhea, nausea, and vomiting, also leading to discontinuation, were the most commonly reported adverse events with cotadutide compared with placebo and liraglutide at any tested dose (160).

The dual GLP-1/glucagon receptor agonist JNJ-64565111 (efinopegdutide) was compared with placebo and liraglutide 3.0 mg in a 26-wk multicenter study in 474 subjects with high-grade obesity (BMI 35–50 kg/m^2^) without diabetes. In the dual agonist treatment arm, doses were 5.0, 7.4, or 10.0 mg, each with no dose escalation. The percent change from baseline in body weight at *week 26* was significantly greater in all JNJ-64565111 treatment groups and the liraglutide group compared with the placebo group (weight loss ≥5% and ≥10% from baseline, *P* < 0.001). One hundred thirty-one participants in the JNJ-64565111 discontinued treatment (22.2%), due to gastrointestinal adverse effects that increased in frequency and severity with increasing dose of JNJ-645111 (161).

In another randomized controlled trial, the dual GLP-1/glucagon receptor agonist JNJ-64565111 (efinopegdutide) was compared at doses of 5.0 mg, 7.4 mg, or 10.0 mg to placebo for 12 wk in 195 subjects with type 2 diabetes mellitus (A1c 6.5%–9.5%), and obesity (BMI 35–50 kg/m^2^). At the end of the study, no significant reduction in A1c and fasting blood glucose was observed in subjects with obesity and type 2 diabetes treated with JNJ-64565111 compared with placebo. All three doses of JNJ-64565111 significantly reduced body weight compared with the placebo (*P* < 0.001). Most interruptions were associated with gastrointestinal disorders that increase in frequency and severity with rising doses of JNJ-64565111 (162).

In a randomized placebo-controlled, multicenter phase 1 study, the efficacy and safety of the dual GLP-1/glucagon receptor agonist mazdutide (IBI362) at doses of 9 mg and 10 mg weekly were evaluated in subjects with overweight and obesity. The study was for 12 wk in the 9-mg cohort and was for 16 wk in the 10-mg cohort. Both doses of mazdutide significantly reduced weight compared with placebo (mean percent change from baseline −11.7% vs −1.8% in the 9-mg group: *P* = 0.0002; and mean percent change from baseline 9.5% vs. −3.3% in the 10 mg group; *P* = 0.024). Predominantly, gastrointestinal adverse effects were observed in all subjects treated with mazdutide (100%) and in 87.5% of those treated with placebo. None resulted in discontinuation of treatment (163).

## EFFICACY OF GLP-1R-GIPR-GCGR TRI-AGONISM IN CLINICAL STUDIES

The safety and tolerability of multiple and ascending doses of the triple receptor agonist of GLP-1, GIP, and glucagon LY3437943 was evaluated in 72 subjects with type 2 diabetes during 12 wk of treatment in a phase 1 double-blind, randomized, multicenter study. Participants were divided in five groups taking LY3437943 at 0.5 mg (*n* = 9); 1.5 mg (*n* = 9); 3 mg (*n* = 11); 3/6 mg (*n* = 11) 3/6/9/12 mg (*n* = 12) and compared to two a group taking placebo (*n* = 15) and dulaglutide 1.5 mg weekly (*n* = 5) respectively. Mainly gastrointestinal adverse effects occurred in 63%, 60%, and 54% of subjects who received LY3437943, dulaglutide 1.5 mg, and placebo, respectively. In LY3437943-treated subjects compared with placebo, glycated hemoglobin was significantly reduced at doses of 3 mg (A1c −1.4% [90% CI −2.17 to −0.56]), at doses 3/6 mg (A1c −1.6% [90% CI −2.37 to −0.75]), and at doses 3/6/9/12 mg (A1c −1.2% [90% CI −2.05 to −0.45]). Compared with placebo, the statistically significant and most relevant weight loss with LY3437943 was observed in the highest dose group (–8.96 kg [90% CI –11.16 to –6.75]). No significant reductions in weight compared with placebo were observed in subjects treated with dulaglutide 1.5 mg (164).

SAR441255, a synthetic peptide agonist of the GLP-1, GCG, and GIP receptors, structurally based on the exendin-4 sequence. SAR441255 displays high potency with balanced activation of all three target receptors. In animal models, metabolic outcomes were superior to results with a dual GLP-1/GCG receptor agonist. Preclinical in vivo positron emission tomography imaging demonstrated SAR441255 binding to GLP-1 and GCG receptors. In healthy subjects, SAR441255 improved glycemic control during a mixed-meal tolerance test and impacted biomarkers for GCG and GIP receptor activation. Single doses of SAR441255 were well tolerated. The results demonstrate that integrating GIP activity into dual GLP-1 and GCG receptor agonism provides improved effects on weight loss and glycemic control while buffering the diabetogenic risk of chronic GCG receptor agonism (165).

A subsequent double-blind randomized controlled trial evaluated the effect on weight loss of LY3437943 (referred to as retatrutide) at initial doses of 1 mg, 4 mg, 8 mg, and 12 mg in a population of 338 obese or overweight subjects for a follow-up of 48 wk. At the end of the study, there was an 8.7% weight loss in the 1 mg group, a 17.1% weight loss in the 4 mg group, a 22.8% weight loss in the 8 mg group, and a 24.2% weight loss in the 12 mg group compared with a 2.1% weight loss in the placebo group. Most side effects were gastrointestinal (nausea, diarrhea, vomiting, and constipation) and dose related (166).

## CONCLUSIONS

Clinical efficacy studies of several unimolecular dual and tri-agonists targeting the GLP-1R, GIPR, and GCGR have demonstrated favorable results both as monotherapies and when combined with approved hypoglycemics. It is not known whether additional synergistic or antagonistic interactions among these G-protein receptor signaling pathways arise from simultaneous receptor activation at the islet cells. The signaling pathways affected by dual- and tri-agonism require more further and more detailed investigation before a comprehensive elucidation of their cellular actions is possible. These investigations will be crucial for understanding the chronic efficacy and long-term safety of these treatments.

## GRANTS

F. Folli was funded by Departmental Line 2-PSR 2021—Department of Health Science, University of Milan, Milano, Italy.

## DISCLOSURES

No conflicts of interest, financial or otherwise, are declared by the authors.

## AUTHOR CONTRIBUTIONS

F.F., R.M., A.G. C.P., and P.B.H. conceived and designed research; F.F., G.F., A.G., F.C., L.C. C.P., and P.B.H. performed experiments; C.B., P.F., and A.D. analyzed data; F.F., P.B.H., G.F., A.G., F.C. and L.C. interpreted results of experiments; F.F., G.F., A.G., F.C., L.C., C.P., and P.B.H. prepared figures; F.F., C.P., R.M., A.G., and P.B.H. drafted manuscript; F.F., R.M., A.G., C.B., P.F., A.D., S.L.R., C.P., and P.B.H. edited and revised manuscript; F.F. and P.B.H. approved final version of manuscript.
